# Squamous–columnar junction of Von Ebner’s glands may be a significant origin of squamous cell carcinomas in the base of the tongue

**DOI:** 10.3389/fonc.2022.1029404

**Published:** 2022-11-16

**Authors:** Peng-Ning Chen, Xin-Yu Chen, Guan-Xi Chen, Lin Luo, Qi-Zhang Yan, Ping Ruan, Ping Li, Da-Hai Yu

**Affiliations:** ^1^ Department of Oral and Maxillofacial Surgery, Guangxi Medical University College Of Stomatology; Guangxi Key Laboratory of Oral and Maxillofacial Rehabilitation and Reconstruction; Guangxi Clinical Research Center for Craniofacial Deformity, Nanning, China; ^2^ Department of Pathology, Ruikang Hospital Affiliated to Guangxi University of Chinese Medicine, Nanning, China; ^3^ Department of Pathology, Guangxi Medical University College Of Stomatology, Nanning, China; ^4^ Department of Stomatology, The First Affiliated Hospital of Guangxi Medical University, Nanning, China

**Keywords:** Von Ebner’s glands, TSC22d1, base of tongue (BOT), squamous-columnar junction (SCJ), squamous cell carcinomas (SCCs)

## Abstract

**Objectives:**

The histological origin of base of the tongue (BOT) carcinomas is still elusive, and most studies have been focusing on the lingual tonsil. In this study, we sought to identify the existence of the squamous–columnar junction (SCJ) in the human Von Ebner’s glandular duct and explored the potential of that in forming squamous cell carcinomas in BOT.

**Materials and methods:**

The specific genomes of BOT carcinoma were acquired and screened out by The Cancer Genome Atlas (TCGA) database analysis. The 4-nitroquinoline-1-oxide (4-NQO)-treated mouse model was used to explore the transformation of SCJ during cancerization. We used immunohistochemistry to confirm the characteristics of SCJ in human Von Ebner’s gland, which were further compared with those in the anus and cervix.

**Results:**

The SCJ in the human Von Ebner’s glandular duct was found to be similar to that of the cervix and anus. The transformation zone in the 4-NQO-treated mouse model had a multilayered epithelium structure similar to that of HPV16-transgenic mice. In human, the transformation zone of Von Ebner’s gland is also similar to that of the cervix and anus.

**Conclusion:**

It is the first time that the existence of SCJ in the opening of the human Von Ebner’s glandular duct was confirmed. The SCJ of Von Ebner’s glands may be a significant origin of squamous cell carcinomas in BOT.

## Introduction

Head and neck squamous cell carcinoma (HNSCC) is the sixth largest malignant tumor in the world. In 2020, HNSCC caused more than 800,000 new cancers and 450,000 deaths (1). Oropharyngeal squamous cell carcinoma (OPSCC) accounts for a quarter of total head and neck cancers, showing poor prognosis and high mortality (2). The molecular pathogenesis of OPSCC is complex and is caused by a wide range of events that involve the interplay between genetic mutations, altered levels of transcripts, proteins, and metabolites (3). The risk factors that are closely related to OPSCC include smoking, drinking alcohol, and human papillomavirus (HPV) (4). An increased incidence of OPSCC has been observed in many Western countries, particularly tonsillar squamous cell carcinoma and BOT carcinoma, which are the two main HPV-positive OPSCC sites (5). Patients with HPV-positive cancers are reported to be younger and have better overall survival than those with corresponding HPV-negative cancers. The progression-free survival rate for HPV-positive and HPV-negative cancers at 2 years was 86% versus 53%, and the overall survival rate for those at 2 years was 95% versus 62% (6, 7).

Compared with human oropharynx, the oropharynx of the mouse (including BOT) lacks the Waldeyer ring, which consists of tonsils and other lymphoid tissues (8). This implies that mice lack crypts lined by reticulated squamous epithelium that are susceptible to HPV infection (9). However, in the nude mice infected with mouse papilloma virus (MmuPV) in the dorsal and ventral surfaces of the tongues, squamous cell carcinomas (SCCs) still prefer to develop at the circumvallate papillae in BOT (10). Meanwhile, a large number of BOT carcinomas confined to Von Ebner’s gland of circumvallate papillae were still found in HPV16-transgenic mice where the expression of all the HPV16 early genes is targeted to keratinizing squamous epithelia by the cytokeratin 14 (Krt14) gene promoter (11). An SCJ exists in the terminal ducts of Von Ebner’s glands of the mouse BOT, which are susceptible to the formation of non-keratinized SCC in the presence of MmuPv or HPV infection. Similarly, in our 4-nitroquinoline-1-oxide (4-NQO)-treated mouse model, the BOT is also a favored site for the development of keratinized SCC (12).

Von Ebner’s gland, which is under the vallate papilla, is a serous gland, and it contributes to taste by secreting fluids from the opening of its ducts located at the bottom of vallate papilla (13). In the past decade, there is no study about human cancerization on this site. However, there was a study showing that CK7-positive SCCs derived from the salivary gland ductal epithelium were present in human BOT carcinomas (14). As a widely accepted marker of transformation zone, CK7 is broadly expressed within the transformation zone between different types of epithelium (15).

At present, the transformation zone between different types of epithelium is recognized as the hotspots of precancerous lesions and carcinomas (16). In the cervix and anus, SCJ is closely related to HPV-induced cervical carcinomas and rectal carcinomas (17). In this area, a transformation zone will arise after the process of squamous metaplasia by local carcinogenic factors. Moreover, the transformation from SCJ to carcinoma is not restricted to HPV infection. In the esophagus, the stimulation of gastric acid in reflux esophagitis can cause Barrett’s esophagus (BE) in the gastro-esophageal junction, and then BE will turn into esophageal carcinoma under continuous stimulations (18). All of these studies hint that Von Ebner’s glandular duct may have a vulnerable region in which malignant transformation of SCC may occur not only in the presence of HPV (non-keratinized SCC), but also in the presence of local carcinogenic factors such as tobacco (keratinized SCC).

In this study, by using TCGA data, the RNA-seq transcriptome information of BOT carcinoma was compared with that of oral tongue carcinoma and tonsillar carcinoma. We screened out the specific genome of BOT carcinoma, and then we used the specific gene product of BOT carcinomas, columnar epithelium marker CK7, and squamous epithelium marker CK5 to compare the SCJ of human vallate papillae with that of the cervix and anus by immunohistochemistry. After that, we confirmed the existence of SCJ where squamous metaplasia can develop at the opening of Von Ebner’s gland and studied its features.

## Materials and methods

### Genomic datasets

The RNA-seq transcriptome information for three types of SCCs (BOT carcinoma, oral tongue carcinoma, and tonsillar carcinoma) was obtained from The Cancer Genome Atlas (TCGA) Data Portal (https://tcga-data.nci.nih.gov). Twenty BOT carcinomas, 134 oral tongue carcinomas, and 40 tonsil carcinomas were included in the analyses.

### Selection of differential genes

To select genes that were differentially expressed between three types of SCCs, two-sample *t*-tests were performed for two combinations of the three carcinoma groups (BOT carcinoma and oral tongue carcinoma; BOT carcinoma and tonsillar carcinoma). *p* < 0.001 was set as a stringent significance cutoff value and there had to be at least a 1.5-fold difference. A heatmap was generated using the R programming language. These two datasets of upregulated differential genes were analyzed using the Venn diagram, and the upregulated genes shared by these two datasets were used as the specific genes of BOT carcinoma. Then, protein coding genes were picked out from the specific genes to make further experimental study. All statistical analyses were performed using the R language environment (http://www.r-project.org).

### Sample collection

Paraffin samples of 10 cases with tongue squamous cell carcinoma that had been excised in surgery and diagnosed by pathology between July 2020 and March 2021 from the Affiliated Stomatological Hospital of Guangxi Medical University were selected, and the vallate papillae of normal tissues around the carcinomas were resected for further experimental study. Six anal paraffin samples resected by internal hemorrhoidectomy and six cervical paraffin samples resected by hysterectomy were selected between 2021 and 2022 from Ruikang Hospital Affiliated to Guangxi University of Chinese Medicine. All the specimens were routine serial sections with a thickness of 5 μm.

### Immunohistochemistry for TSC22D1, CK7, and CK5

For immunostaining, the sections were dewaxed, hydrated in alcohol, washed three times in distilled water, heated in a pressure cooker in Tris-EDTA (pH 9.0) to retrieve antigenic activity, and then cooled at room temperature. Endogenous peroxidase activity was inhibited by incubation with 3% hydrogen peroxide for 20 min at 37°C. After nonspecific reactions had been blocked, the sections were incubated overnight at 4°C with the following primary antibodies: CK5 (OTI1C7, 1:200; ZSGB-BIO, Beijing, China) and CK7 (EP16, 1:200; ZSGB-BIO, Beijing, China). The sections were then incubated with biotinylated goat anti-mouse immunoglobulin G (IgG) for 20 min at 37°C. Careful rinses were performed with several changes of PBS buffer between each stage of the procedure. Finally, the color was developed with diaminobenzidine and then the sections were counterstained with hematoxylin.

The immunohistochemistry procedure for TSC22D1 (Q15714, 1:150; ABmart, Shanghai, China) is much the same as that of CK7 and CK5 except that antigenic activity needs to be retrieved using citrate buffer (pH 6.0). Pathologists are responsible for the diagnosis.

### Animals and carcinogen treatment

Six-week-old male BALB/c mice, which were obtained from and raised at the Guangxi Medical University Laboratory Animal Center, were used in the present study. The mice were handled in accordance with the Animal Care and Use Guidelines of Guangxi Medical University, and the study protocol was approved by the Institutional Animal Care and Use Committee. The experiments were carried out under controlled conditions including a 24-h light/dark cycle, and the mice were maintained in a room with a relative humidity level of 30%–50% and a temperature between 18°C and 25°C. The mice were fed standard mouse chow. All mice were acclimatized to the laboratory environment for 1 week before the experiments were begun. The carcinogen 4-NQO (Sigma-Aldrich, St. Louis, MO, USA) was prepared at a concentration of 0.1% w/v in drinking water and stored at 4°C. The 4-NQO stock solution was diluted to a concentration of 200 lg/ml in the drinking water, and the water was replaced once a week. The mice were allowed access to the drinking water and chow diet during the treatment.

### Statistical analysis

Genes with Log2|fold change (FC)| > 1.5 and FDR < 0.01 were found to be differentially expressed and were significantly enriched.

## Results

### Identification of the specific genes in BOT carcinoma

**Figure 1:**
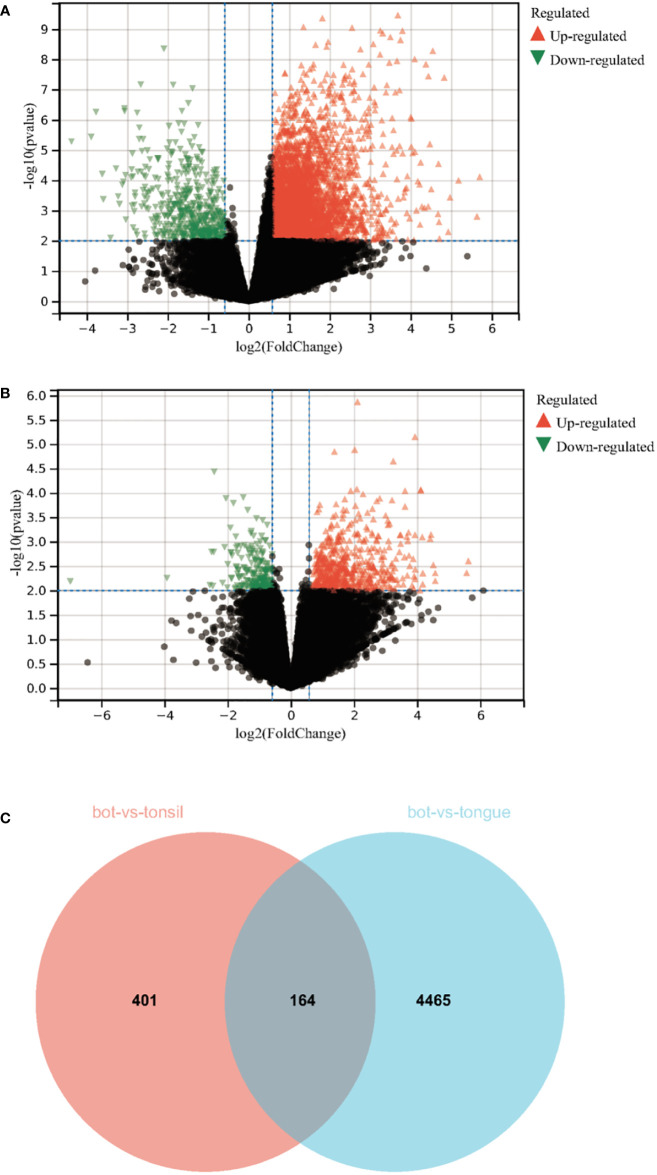
Identification of the specific genes in BOT carcinoma. **(A)** The volcanic map of the differentially expressed genes between BOT carcinoma and oral tongue carcinoma. **(B)** The volcanic map of the differentially expressed genes between BOT carcinoma and tonsillar carcinoma. **(C)** A total of 164 upregulated genes of BOT carcinoma in oral tongue carcinoma and tonsillar carcinoma were analyzed by Veen diagram. Genes with Log2|fold change (FC)| > 1.5 and FDR < 0.01 were found to be differentially expressed and had significant enrichment.

To explore the specific genes in BOT carcinoma, all RNA expression data for three types of SCCs (BOT carcinoma, oral tongue carcinoma, and tonsillar carcinoma) were downloaded to analyze the differentially expressed gene in BOT carcinoma. Genes with Log2|fold change (FC)| > 1 and FDR < 0.05 were found to be differentially expressed and were significantly enriched. The volcanic map showed 565 upregulated differential genes and 211 downregulated differential genes in the BOT carcinoma *vs*. tongue carcinoma group (**Figure 1A**). The volcanic map showed 4,629 upregulated differential genes and 510 downregulated differential genes in the BOT carcinoma *vs*. tonsillar carcinoma group (**Figure 1B**). The 164 common upregulated genes were identified in the BOT carcinoma *vs*. tongue carcinoma group and the BOT carcinoma *vs*. tonsillar carcinoma group (**Figure 1C**). Nine protein-coding RNAs were discovered (**Table 1**; **Supplementary Material**); HCRTR2, TSC22D1, RGPD5, TBX20, TAS1R2, LYC22D1, AC008764.4, and AC008758.5 are the specific genes of BOT carcinoma. Meanwhile, a research showed the identification of the TSC22D1 as a specific gene of BOT carcinoma (19). Hence, we finally chose TSC22D1 as a specific gene of BOT carcinoma to carry out the next phase of research.

**Table 1:** The 164 common up-regulated genes were identified in BOT carcinoma vs tongue carcinoma group and BOT carcinoma vs tonsillar carcinoma group.

Gne name	RNA type
AC020661.2	lncRNA
SYNGR2P1	Processed pseudogene
AC091152.1	Processed pseudogene
AL157778.1	lncRNA
HCRTR2	Protein coding
AL023775.1	Processed pseudogene
TSC22D1	Protein coding
RPL23AP52	Processed pseudogene
RPS15AP24	Processed pseudogene
AC188616.1	lncRNA
AC012085.2	lncRNA
AL513302.1	Processed pseudogene
PPY2P	Transcribed unprocessed pseudogene
AC025259.2	Processed pseudogene
AC211433.2	lncRNA
AL513533.1	Processed pseudogene
ETF1P3	Processed pseudogene
RPL31P61	Processed pseudogene
RPL36AP45	Processed pseudogene
AC093330.1	lncRNA
LINC01488	lncRNA
KATNBL1P6	Transcribed processed pseudogene
AL031133.1	Processed pseudogene
AL121990.1	lncRNA
AC021218.1	lncRNA
AL589765.3	Processed pseudogene
RNU12-2P	snRNA
AC025887.2	lncRNA
SRP14P1	Processed pseudogene
PGAM1P10	Processed pseudogene
LRRC37A12P	Unprocessed pseudogene
RBMX2P3	Processed pseudogene
SLC6A12-AS1	lncRNA
AC093627.22	lncRNA
RGPD5	Protein coding
AC139792.1	lncRNA
SKP1P2	Processed pseudogene
AC006511.1	Unprocessed pseudogene
TBX20	Protein coding
AC008764.4	Protein coding
PPIAP86	Processed pseudogene
AC046176.1	Processed pseudogene
AC096992.3	lncRNA
AC068790.9	lncRNA
RN7SL515P	misc RNA
AC090403.1	lncRNA
ECEL1P2	Transcribed unprocessed pseudogene
RPS3AP39	Processed pseudogene
AC105415.1	lncRNA
TAS1R2	Protein coding
EIF5P1	Processed pseudogene
AC095057.1	Processed pseudogene
AC018761.3	lncRNA
AC114485.1	lncRNA
AC188617.1	lncRNA
KRT18P35	Processed pseudogene
AC008892.1	lncRNA
AC108676.2	Processed pseudogene
AC107959.2	lncRNA
AC005307.1	lncRNA
AC108868.1	lncRNA
AC013467.1	Processed pseudogene
AC119673.1	lncRNA
AC006398.1	lncRNA
AC061975.5	Unprocessed pseudogene
MTCYBP21	Processed pseudogene
AL606970.3	lncRNA
PPIAP15	Processed pseudogene
LYZL2	Protein coding
DPRXP6	Processed pseudogene
FAM189A2	Protein coding
CLDN4	Protein coding
AC127496.4	lncRNA
AC096644.3	lncRNA
SSBL3P	Processed pseudogene
LINC02232	lncRNA
AC022148.2	lncRNA
AL121970.1	lncRNA
AC104260.3	lncRNA
AC023421.2	lncRNA
CDRT15P14	Unprocessed pseudogene
AL133297.2	lncRNA
NPM1P34	Processed pseudogene
AL360007.1	lncRNA
AC117465.1	lncRNA
AC011676.3	lncRNA
AC099336.1	Transcribed processed pseudogene
AP000696.1	lncRNA
VN1R51P	Processed pseudogene
AC131212.1	lncRNA
MIR548A3	miRNA
POM121L10P	Processed pseudogene
AC239860.4	lncRNA
AC066616.1	Processed pseudogene
AC113367.3	Processed pseudogene
CCT5P1	Processed pseudogene
SUMO2P3	Processed pseudogene
ARPP19P1	Processed pseudogene
AC082651.1	lncRNA
AC008525.1	lncRNA
AC007652.2	lncRNA
RPS7P4	Processed pseudogene
AP003168.2	lncRNA
AL449363.1	Processed pseudogene
LINC02441	lncRNA
LINC02197	lncRNA
AC092640.1	lncRNA
AC026353.1	lncRNA
AC069335.1	Processed pseudogene
RN7SL711P	misc RNA
AC018563.1	lncRNA
PRSS52P	Unprocessed pseudogene
AL031121.3	lncRNA
AL133343.1	lncRNA
LINC00559	lncRNA
AC064869.1	lncRNA
AC005906.1	Processed pseudogene
ADI1P2	Processed pseudogene
RNU6-540P	snRNA
AC104640.1	lncRNA
AC010480.1	TEC
AC018450.2	lncRNA
MTCO3P40	Processed pseudogene
OR7E2P	Unprocessed pseudogene
AC006960.1	Processed pseudogene
RPSAP59	Processed pseudogene
GJA6P	Processed pseudogene
HMGB1P46	Processed pseudogene
NUDT19P6	Processed pseudogene
FP325330.1	lncRNA
AL499627.2	lncRNA
LINC02673	lncRNA
CYP4F36P	Unprocessed pseudogene
AC055717.3	TEC
AC016598.2	lncRNA
AL139390.1	lncRNA
MED6P1	Processed pseudogene
AL079307.2	lncRNA
AC117945.2	lncRNA
MTND2P29	Processed pseudogene
CLIC4P2	Processed pseudogene
MTND1P3	Processed pseudogene
AC079790.1	lncRNA
RNU6-1154P	snRNA
UGT1A12P	Unprocessed pseudogene
PGAM4P1	Processed pseudogene
TARM1	Protein coding
AL360268.2	lncRNA
RN7SL131P	misc RNA
CLYBL-AS1	lncRNA
RN7SL706P	misc RNA
AC004790.1	Unprocessed pseudogene
LINC01056	lncRNA
DUX4L47	Processed pseudogene
AC008758.5	Protein coding
AP000146.1	lncRNA
AC007652.3	lncRNA
AC091905.4	TEC
AL035443.1	lncRNA
MIR551A	miRNA
AC091163.3	Processed pseudogene
AL109829.1	lncRNA
AL157687.1	Processed pseudogene
AP001625.1	lncRNA

### Observation of BOT in the 4-NQO-treated mouse model

**Figure 2:**
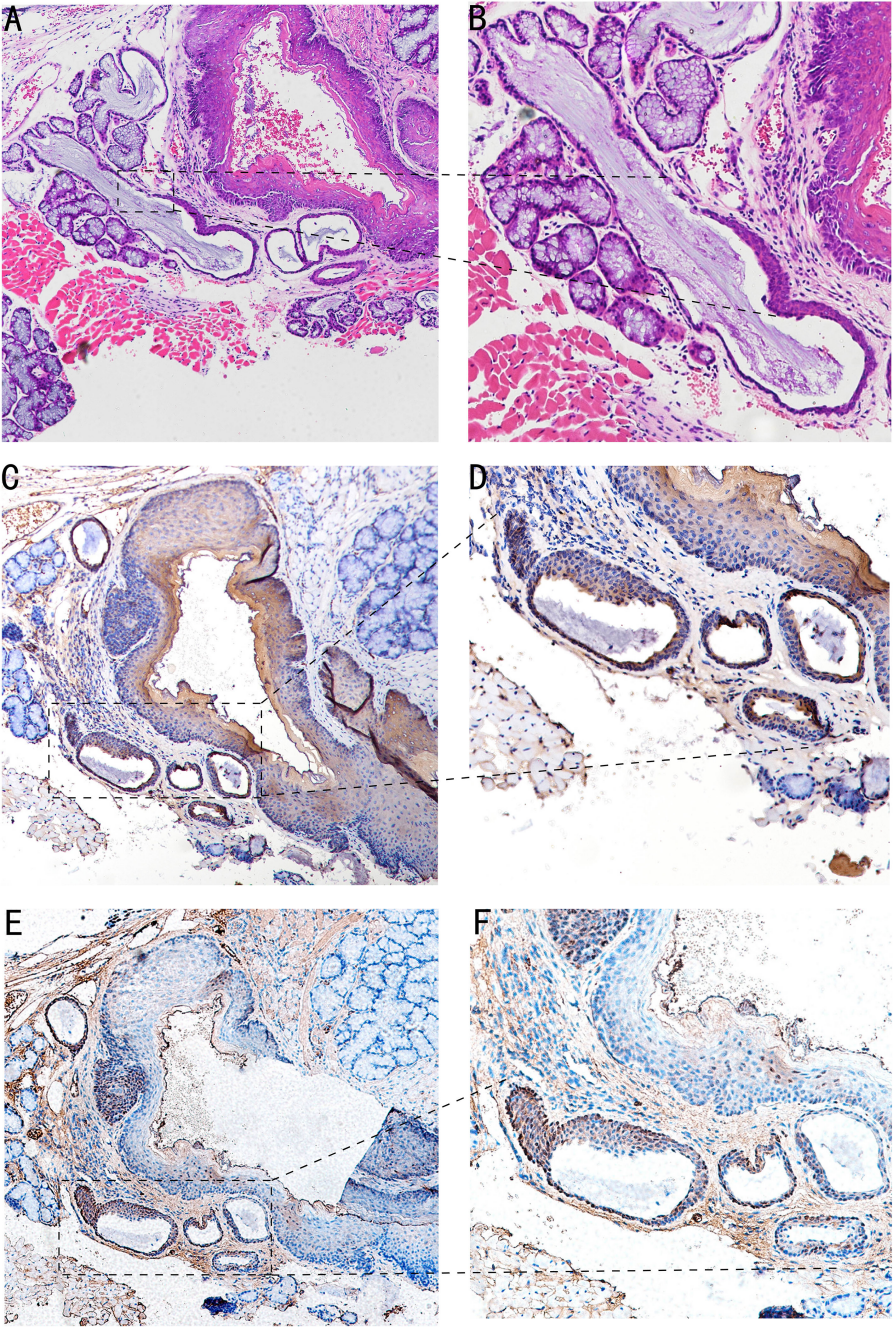
Image of BOT in the 4-NQO-treated mouse model of oral squamous cell carcinomas at 30 weeks. **(A, B)** HE staining of Von Ebner’s glandular duct in mouse BOT; the dotted line denotes the transformation zone (A,×4; B,×10). **(C, D)** The CK7 expression of Von Ebner’s glandular duct in mouse BOT; dotted line can indicates squamous metaplasia and dilatation of branching ductus (C,×4; D,×10). **(E, F)** The TSC22D1 expression of Von Ebner’s glandular duct in mouse BOT; the dotted line denotes squamous metaplasia and dysplasia of ductal epithelium, and rarely positive in complete squamous metaplasia of the anterior segment of ductal epithelium.TSC22D1 was consistently positive in the dysplasia of ductal epithelium (**E**,×4; **F**,×10).

In the 4-NQO-treated mouse model, we found that abnormal hyperplasia of the circumvallate papilla could appear in BOT at 30 weeks. HE staining showed that Von Ebner’s glandular ducts were tortuous and expanded, the crypts were significantly deepened, and squamous metaplasia occurred at the opening (**Figures 2A, B**). It can be seen that CK7 is weakly expressed in proliferative epithelial cells (**Figures 2C, D**). Furthermore, TSC22D1 is weakly expressed in the anterior segment of the completely squamous metaplasia ductal epithelium, and TSC22D1 is strongly positive in dysplastic epithelium and normal ductal epithelium (**Figures 2E, F**).

### Observation in Von Ebner’s gland, cervix, and anus with HE staining

**Figure 3:**
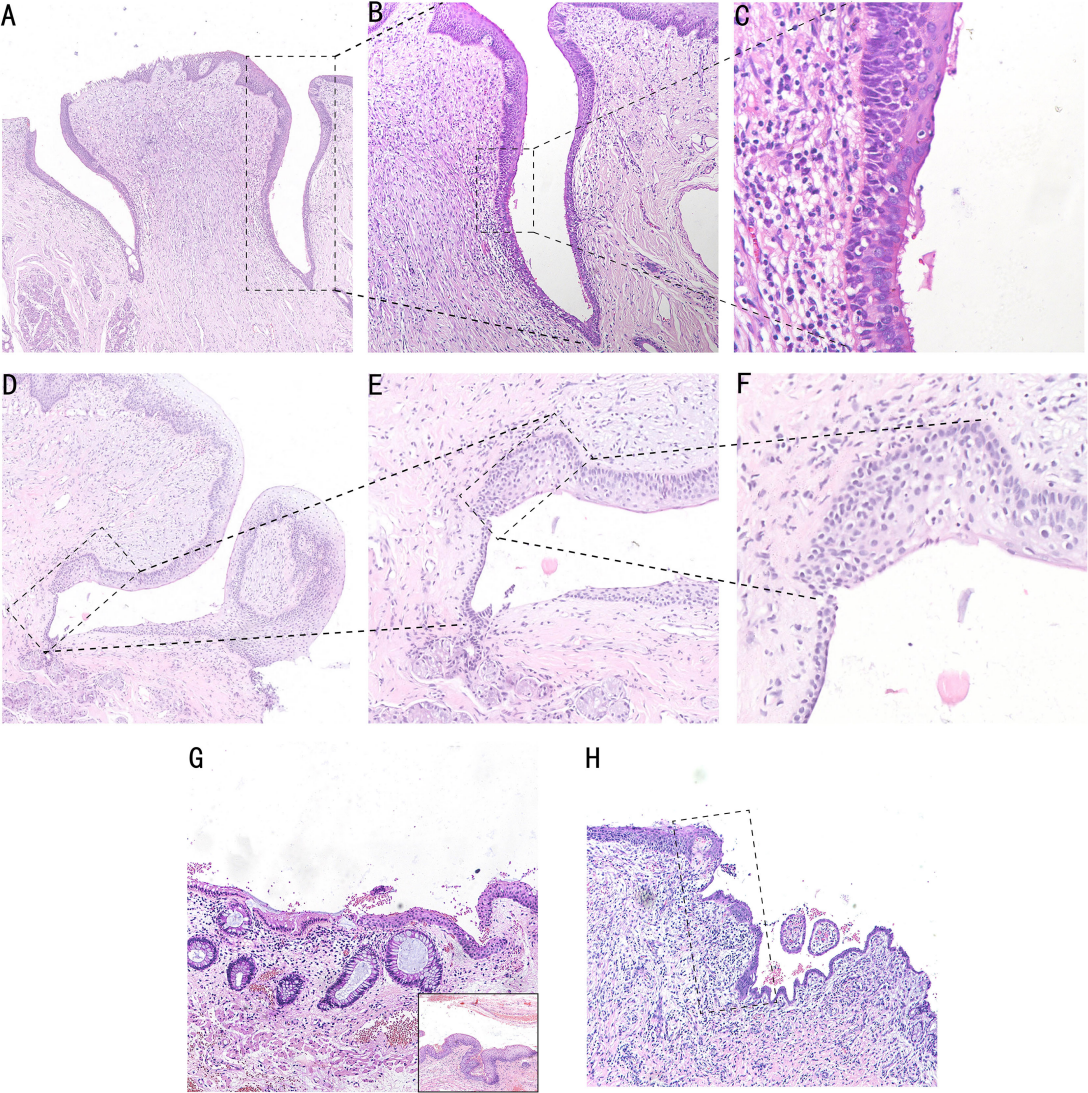
HE staining in the human cervix, anus, and Von Ebner’s gland. The SCJ in the opening of Von Ebner’s gland. Pseudostratified ciliated columnar epithelium of the columnar area and stratified squamous epithelium of the squamous area can be seen (**A**, ×5; **B**, ×10; **C**m ×20). **(D–F)** HE staining of squamous metaplasia SCJ of Von Ebner’s gland, and transitional area hyperplasia can be seen in the dotted line area, which is shown as a multilayered epithelium (**D**, ×4; **E**, ×10; **F**, ×20). **(G)** HE staining of anal SCJ, and the multilayered epithelial structure of the transitional zone can be seen. In the bottom right section of the image, the junction of the transitional zone with the squamous epithelial is shown (**G**, ×4). **(H)** HE staining of cervical SCJ, and the dotted line area is the transitional zone with a monolayer epithelial structure (**H**, ×4).

We found an SCJ between squamous epithelium and columnar epithelium in the opening of Von Ebner’s glandular duct at the bottom of vallate papilla by the HE staining technique. The squamous epithelial zone beside the SCJ consists of seven to eight layers of non-keratinized stratified squamous epithelium, which is gradually in transition to the keratinized squamous epithelium at the top of vallate papilla. The columnar epithelial zone of the duct beside the transitional zone comprised three to four layers of pseudostratified ciliated columnar epithelium. The SCJ is approximately 10–20 cells in size, which consists of ciliated columnar epithelium and stratified squamous epithelium. Intercellular bridges can be seen in stratified squamous epithelium and the transitional zone (**Figures 3A–C**). The transitional area of Von Ebner’s gland with squamous metaplasia is represented by 8–10 layers of multilayered epithelium with a length of 20–30 cells without pseudostratified ciliated columnar epithelium (**Figures 3D–F**).

The reserve cells at the base of the SCJ of the cervix, anal canal, and Von Ebner’s gland differentiate towards the squamous epithelium in the presence of local stimulating factors and form a composite structure with a superficial columnar epithelium and basal squamous epithelium. Both Von Ebner’s gland and the anal transitional zone are multilayered epithelium, whereas the cervix is a single layer of epithelium. Compared with the human Von Ebner’s gland transitional zone, the anal transitional zone is longer and irregular, ranging from 4 to 700 cells in length (17). However, unlike the anal and cervical columnar epithelium, which are both single layered (**Figures 3G, H**), the epithelial type of the columnar epithelial segment of the Von Ebner’s gland is pseudostratified ciliated columnar epithelium (**Figure 3C**). The ciliated columnar epithelial structure of Von Ebner’s gland promotes the excretion of saliva and food residues, and the loss of ciliated-like structures during metaplasia will further impact excretory function.

### Comparative observation in the cervix, anus, and Von Ebner’s gland with IHC

**Figure 4:**
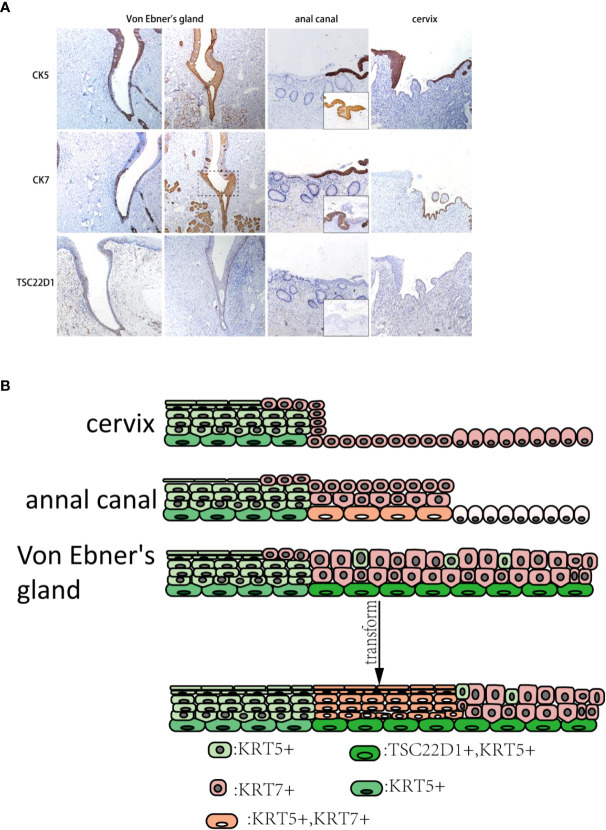
**(A)** CK5, CK7, and TSC22D1 IHC staining in the human cervix, anus, and Von Ebner’s gland. The dashed line is shown in the CK7-positive squamous metaplasia of human Von Ebner’s gland, where CK5 is also expressed. In contrast, normal Von Ebner’s gland has no CK5+/CK7+ multilayered epithelium. TSC22D1 was only expressed in the squamous metaplasia zone and columnar epithelial areas of Von Ebner’s gland. CK5+/CK7+ multilayered epithelium can be seen in the anal SCJ. CK5+/CK7+ squamous metaplastic island can be seen in the cervix (×10). (The lower right of the anal canal diagram shows the junction of the transitional zone with the squamous epithelium.) **(B)** Pattern diagram of the transformation zone in the cervix, anus, and Von Ebner’s gland.

The consecutive expression of CK7 can be seen in taste buds of the squamous area and columnar epithelial area duct segment in Von Ebner’s gland, and the CK7-positive monolayer columnar epithelium was also present in the surface layer of SCJ. CK5 was expressed in the squamous epithelial zone, in the basal layer of the columnar epithelial zone, and in reserve cells in the ductal epithelium. A small number of CK7-positive columnar epithelium can be seen on the surface layer of cervical SCJ with CK5-positive multilayered squamous epithelium under. However, CK5/CK7-positive multilayered epithelia were found in the islands of squamous epithelium where metaplasia occurred. Similarly, after metaplasia occurred in Von Ebner’s gland, the transition zone showed CK5/CK7-positive multilayered epithelium. Compared to normal Von Ebner’s gland, CK5 was expressed in the squamous area and the transformed zone of the metaplastic Von Ebner’s gland. In addition, tsc22d1-positive basal cells were seen consecutively in the basal layer of the columnar epithelial and transition zones of Von Ebner’s gland, whereas there were no tsc22d1-positive basal cells in the cervix and anal canal (**Figures 4A, B**).

## Discussion

The insidious onset and early lymphatic metastasis of BOT carcinoma result in advanced stage and poor prognosis when diagnosed (20). The difficulty of early diagnosis makes it hard to confirm the histological origin of BOT carcinoma clinically. Tongue tonsils have crypt structures that are similar to palatine tonsils; thus, they are also the predilection sites of HPV-induced HNSCC (21). According to the results of gene data analysis of BOT carcinoma and palatine tonsillar carcinoma, BOT carcinoma has almost the same genome as tonsillar carcinoma (19); the lingual tonsil has always been a research priority on the origin of BOT carcinoma. However, it has been reported that some BOT carcinomas are deep-seated and originate from the large excretory duct of the submucosal minor salivary gland. This BOT carcinoma lacks obvious surface components, and salivary ducts adjacent to the carcinoma showed extensive intraductal hyperplasia and metaplasia (14). It reminds us that BOT carcinoma may originate from the salivary duct epithelium. Therefore, we speculate that BOT carcinoma has other predilection sites.

In order to find the specific genes of BOT carcinoma, we screened and identified the differential genes of BOT carcinoma, oral tongue carcinoma, and tonsillar carcinoma in TCGA open datasets, and finally picked out TSC22D1 as the specific gene of BOT carcinoma. TSC22D1 is one of TSC22 domain family members encoding leucine zipper transcription factor (22), and it can be expressed in salivary adenocarcinoma and normal ductal epithelium. TSC22D1 can negatively regulate the development of salivary adenocarcinoma cells and have the function of maintaining and regulating the differentiation of salivary gland cells. TSC22D1 is weakly expressed in cells that have growth potential, but strongly expressed in cells with differentiated phenotypes (23). A population of stem cells at the bottom of trench areas at the base of circumvallate and foliate papillae can rise to taste cells and perigemmal cells (24). TSC22D1 is consistently expressed at sites of epithelial–mesenchymal interactions in the embryonic development of the mouse (25). TSC22D1 is a specific gene in BOT carcinoma and is closely related to the secretory salivary gland; hence, BOT carcinoma may come from Von Ebner’s gland SCJ with a secretory phenotype. In our study, we found that the epithelial hyperplasia and squamous metaplasia areas have weak expression of TSC22D1 in Von Ebner’s gland’s transformation zone, but it has strong expression in dysplastic epithelial cells of the anterior segment of Von Ebner’s glandular duct in mice. We speculated that the expression of TSC22D1 may be related to the differentiation potential of cells in Von Ebner’s gland, and it was inhibited in the process of squamous metaplasia.

Our study found that the squamous metaplasia of Von Ebner’s glandular duct exists in the 4-NQO-treated mouse model; analogous squamous metaplasia was also found in serial sections of different human Von Ebner’s glands. Compared with the cervix and anus, TSC22D1 was only expressed in the basal layer of the transformation zone in Von Ebner’s gland and expression was maintained after squamous metaplasia. A large number of studies have shown that metaplasia in the transformation zone of different organs can develop into SCCs under the influence of HPV infection and other factors (26, 27). The transition zone showed positive CK5/CK7 in the cervix, esophagus, and anus (17, 28).

In the cervix and esophagus, one of the origins of transition zone cells has been derived from residual CK7+embryonic cells (28, 29). Compared to other salivary glands, Von Ebner’s glands have taste stem cells in the SCJ. In recent studies, it has been revealed that there is the presence of a small population of Lgr5+ cells at the base of the circumvallate and foliate papillary sulcus with stem cell functions that can differentiate into taste and epithelial cells (24). Lgr5 was proved to be expressed in gastrointestinal crypt stem cells and can also be used to identify tumor stem cells in colon cancer (30, 31). Hence, we hypothesize that such Lgr5+ cells can also become cancerous under local stimulus factors like HPV and smoking.

Unlike the cervix, HPV-associated precursor lesions in the oropharynx have not been identified (32), which may be related to the lack of awareness of the pathology of Von Ebner’s gland due to its insignificant precursor lesions, which poses a challenge for early diagnosis and screening. The SCJ region of Von Ebner’s gland opening was found to be the primary site of HPV-associated SCC in a study of HPV mice (11). However, the cancerization potential of this region in the 4-NQO-treated mouse model (12) as well as in humans has not been demonstrated. Our study identified precursor lesions in Von Ebner’s gland that are associated with tobacco and alcohol stimulation factors in humans and mice, which has prospective implications for the study of early lesions of BOT cancer.

One limitation of this study is that it is only a serial section study in which immunohistochemistry was conducted, but no immunofluorescence multiple staining was performed. There is no in-depth study of transformation zone epithelium by genetic lineage tracing in cytologic and animal models. Further research in embryological and animal models is needed in the future.

## Data availability statement

The datasets presented in this study can be found in online repositories. The names of the repository/repositories and accession number(s) can be found in the article/**Supplementary Material**.

## Ethics statement

The animal study was reviewed and approved by Institutional Animal Care and Use Committee of Guangxi Medical University.

## Author contributions

Contribution Study concepts: P-NC. Study design: D-HY. Quality control of data and algorithms: X-YC. Data analysis and interpretation: G-XC, LL. Statistical analysis: Q-ZY. Manuscript preparation: P-NC, X-YC. Manuscript editing: PR, PL. Manuscript review: D-HY. All authors contributed to the article and approved the submitted version.

## Funding

This study was supported by the Natural Science Foundation of China (Grant No. 81360407) and the Guangxi Natural Science Foundation (Grant No. 2016GXNSFDA380002). The funders had no role in the study design, data collection and analysis, decision to publish, or manuscript preparation.

## Acknowledgments

The authors gratefully acknowledge all the study participants and study staff for their help and cooperation during this study.

## Conflict of interest

The authors declare that the research was conducted in the absence of any commercial or financial relationships that could be construed as a potential conflict of interest.

## Publisher’s note

All claims expressed in this article are solely those of the authors and do not necessarily represent those of their affiliated organizations, or those of the publisher, the editors and the reviewers. Any product that may be evaluated in this article, or claim that may be made by its manufacturer, is not guaranteed or endorsed by the publisher.
